# Improvement in the definition of anti-HLA antibody profile in highly sensitized patients

**DOI:** 10.1371/journal.pone.0171463

**Published:** 2017-02-03

**Authors:** Juan Irure, Esther Asensio, Emilio Rodrigo, Íñigo Romón, Javier Gómez, Manuel Arias, Marcos López-Hoyos, David San Segundo

**Affiliations:** 1 Immunology Service, University Hospital Marqués de Valdecilla-IDIVAL, Santander, Spain; 2 Tissue Typing Laboratory, University Hospital Marqués de Valdecilla, Santander, Spain; 3 Nephrology Service, University Hospital Marqués de Valdecilla-IDIVAL, Santander, Spain; 4 Pathology Service, University Hospital Marqués de Valdecilla-IDIVAL, Santander, Spain; Instituto Nacional de Ciencias Medicas y Nutricion Salvador Zubiran, MEXICO

## Abstract

The definition of anti-HLA antibody profile in highly sensitized patients on a waiting list is crucial when virtual crossmatch is used in organ allocation systems, but also when used to identify the true deleterious anti-HLA antibodies. Here we propose different levels of risk based on the results of anti-HLA antibody testing in neat serum (N) and after sera dilution (DIL) and C1q test in 18 highly sensitized patients. This group was heterogeneous in terms of anti-HLA antibody titers and their ability to fix complement. After dilution, 15 out of 18 patients (83.3%) showed a reduction of positive bead counts whereas 4 patients showed a prozone effect and complement fixation was demonstrated. The high dilution of sera and ascertaining the complement fixation allow the accurate definition of risk anti-HLA antibody profiles in highly sensitized patients, as demonstrated in 5 of the sensitized patients who were transplanted.

## Introduction

The anti-HLA antibody testing was revolutionized by solid-phase assays (SPA) based on Luminex, increasing the sensitivity and the ability to detect low levels of anti-HLA antibodies[1]. With Luminex data, unacceptable HLA antigens from potential donors could be assigned and virtual crossmatch (vXM) was addressed[2]. This is especially interesting in the case of highly-sensitized patients because vXM has allowed the development of specific programs to reduce the waiting list for organ transplantation, and an accurate profile of unacceptable HLA antigens should be defined[3].

Despite the improvement of anti-HLA antibody detection by Luminex being well accepted, several questions have risen with the potential pitfalls[4]. When doubtful HLA antigens are considered as unacceptable with mean fluorescence intensities (MFI) near the cut-off value, the knowledge of potential interfering factors should be kept in mind. Two different kinds of errors could appear; a) false positive results: due to a reaction against denatured HLA antigens (neo-epitopes generated during the process of coating the beads with HLA antigens), or due to the lack of consensus about cut-off values to determine positive or negative specificities explained in part to wide intra- and inter-laboratory variability[5]. A significant example is the assessment of unexpected anti-HLA antibodies in non-sensitized, non-transfused males using different cut-off values[6]; on the other hand b) false negative results: due to the potential interaction of complement with IgG anti-HLA antibodies[7] easily overcome after EDTA or heat treatment[8], or due to a prozone effect which could be solved after dilution of the sera[9,10]. Therefore, the overestimation of unacceptable HLA antigens in a patient could lead to an increased time on the waiting list, and an accurate definition of unacceptable and permitted HLA antigens should be addressed.

There is an increasing interest in a clear definition of the anti-HLA antibody profile due to their use in vXM, but also in donor-pair living donors and highly-sensitized programs in order to allocate an organ with negative vXM results. However, in some undesired situations with positive complement-dependent crossmatchs, a negative vXM result is found [11]. This discordance could be improved with further study of the IgG anti-HLA antibodies profile.

Sometimes the definition of the anti-HLA antibodies profile is difficult due to low-titer anti-HLA antibodies that produce variable results in sequential serum studies. Another situation that must be completely ruled out in highly-sensitized patients is the prozone effect where high titers of antibodies compete for the antigen and are not detected in SPA. For this reason. it is crucial to assign these dubious specificities as unacceptable or permitted antigens. In order to facilitate this assignment, two recent publications have demonstrated different strength of anti-HLA antibodies[9,10] as an approach to better defining the anti-HLA antibodies profile.

Here we propose an anti-HLA rating based on anti-HLA antibody strength and the ability to fix the complement in highly-sensitized patients. Such a rating is validated in complement-dependent cytotoxicity (CDC) and flow cytometry crossmatch (FCXM) against peripheral blood mononuclear cells (PBMCs) from blood donors with HLA specificities which are recognized by the sensitized patients´ sera and is further probed in 5 patients who could be transplanted.

## Materials and methods

The work has been conducted according to the principles expressed by the Declaration of Helsinki and approved by the Ethics Comittee of our institution (Comité Ético de Investigación Clínica de Cantabria). The patient on the waiting list signed their informed consent and the sera collection is registered in ISCIII with number: C.0003580.

### Patients and samples

Serum from 18 highly-sensitized patients, with >98% of calculated panel reactive of antibodies (cPRA) and who did not receive any desensitization treatment with Rituximab or intravenous immunoglobulins, were studied for anti-HLA class-I antibodies by Single Antigen (LABScreen Single Antigen Class-I, One Lambda, Canoga Park, CA) at neat (N) and at a 1/160 serum dilution (DIL) based on a previous report[9] where further dilutions did not find any prozone effect. For identification of fixing-complement anti-HLA antibodies, the C1q test was performed (C1qScreen, One Lambda). A total number of 1,746 class-I antigens on beads were evaluated and raw MFI were compared after N, DIL and C1q assays.

The cut-off value was set at 3000 MFI and every single bead with MFI below the cut off value was considered as negative. The number of beads with a positive result was counted in each condition (N, DIL and C1q).

The main demographic, clinical and immunological parameters of the highly-sensitized patients tested are summarized in Table 1.

**Table 1:** Demographic and clinical parameters of highly-sensitized patients.

Age (years mean, SD^a^)	(54.6, 12.3)
Gender (Female/Male)	(5 / 13)
Renal disease (Glomerular/Interstitial/Systemic/Congenital/Unknown)	(10/4/2/1/1)
Transfusions (No / Yes)	(8 / 10)
cPRA^b^	98%
Dialysis (Hemodialysis/Peritoneal)	(16 / 2)
Number of previous transplants (0/1/2/3)	(2 / 6 / 9 / 1)

^a^SD: Standard deviation

^b^cPRA: calculated Panel Reactive of Antibodies

Five out of the 18 kidney transplant recipients (KTR) could be transplanted with negative CDC but positive FCXM and they were biopsied because of clinical suspicion of antibody-mediated rejection (ABMR) or protocol biopsy. Sera at transplantation from these five recipients were retrospectively studied for anti-HLA antibodies, taking into account the three previously-mentioned conditions (N, DIL, C1q) and the risk for rejection was scored/caluclated.

### Complement-dependent cytotoxicity crossmatch

Crossmatch was performed by CDC. Donors´ lymphocytes were separated from whole blood by Ficoll gradient. Cells were suspended in phosphate buffer saline (PBS) to achieve a concentration of 2x10^6^/mL. 1ul of patients’ serum was dispensed in the wells of an oiled Terasaki plate. ½ and ¼ serum dilutions were also used. All samples and dilutions were in duplicate. Negative and positive control samples were dispensed in the same way. Then, 1ul of lymphocytes and 5ul of rabbit complement (One Lambda) were dispensed in each well. After incubation (90 minutes, 22°C), eosin was added to each well and the plate was read in an inverted microscope.

### Flow cytometry crossmatch

PBMC isolated by Ficoll gradient were incubated with pronase during 20 minutes in a 37°C water bath. Neat and 1/160 diluted serum was added to pronase-treated PBMC (Sigma Aldrich, St. Louis, MO) and the mix was incubated during 30 minutes at room temperature. Anti-CD3 Pacific Blue (clone UCHT-1, Immunostep, Salamanca, SPAIN), CD19 APC (clone SJ25C1, BD Biosciences, San Jose, CA) and subsequently Fab´-IgG FITC (Dako, Glostrup, DENMARK) were added and samples were acquired using a FACS-Canto II (BD Biosciences). Flow cytometry crossmatch for class-I antigens was considered positive in the CD3+ gate when the ratio: median fluorescence value Serum / median fluorescence value Negative Control was > 1.5; for class-II antigens, the FCXM was considered positive in CD19+ gate when the ratio was > 2.0.

### HLA typing

Donors were typed for HLA locus A, B and DRB1 by low resolution SSP (Life Technologies, Brown Deer, WI).

### Donor selection

Once a donor HLA-typing was confirmed, the anti-HLA antibody profile was assessed for all sera. If one serum was eligible for anti-HLA antibody profile testing, subsequent CDC and FCXM was performed.

### Statistical analysis

The correlation of mean fluorescence intensities among N, DIL and C1q was assessed by the Spearman test. The number of positive beads was compared by the Wilcoxon test and p<0.05 was considered significant. All the tests were performed using GraphPad software version 5.0 (San Diego, CA).

## Results

### Correlation of raw MFI data

**Fig 1:**
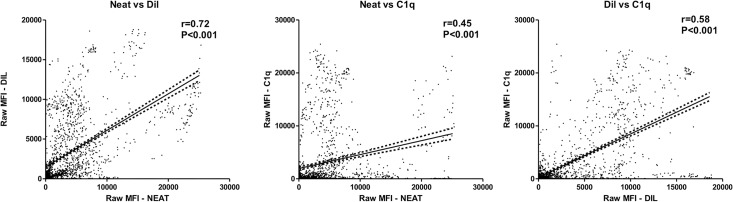
Correlation of raw MFI values after neat, diluted serum and C1q test. The raw MFI value after neat serum (neat), 1/160 diluted serum (DIL) and C1q test (C1q) were compared. The linear regression line and 95% confidence intervals in each plot are depicted (The correlation of MFI values was assessed by Spearman test (***, p<0.001).

In order to assess the relationship of raw MFI data of neat sera (N), diluted (DIL) and C1q test (C1q), the MFI values of 1,746 beads with HLA-class-I antigens were compared (Fig 1). A positive correlation of raw MFI data between N and DIL serum was observed (r = 0.72; p<0.001), whereas the correlation between N and C1q raw MFI values was poorer (r = 0.45; p<0.001). Nevertheless, a better correlation of raw MFI data C1q and DIL serum was observed (r = 0.58; p <0.001).

### Anti-HLA antibody profiles in highly sensitized patients

**Fig 2:**
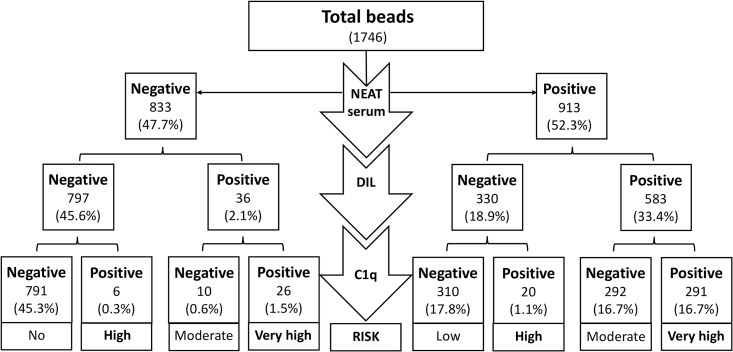
Stratification of the risk regarding positive bead results after neat serum, diluted serum and C1q test. A total number of 1746 beads were distributed according positive (+) and negative (-) results after neat (N), 1/160 diluted (DIL) serum and C1q test. The combination of the results render 8 profiles with potential different risk. The absolute number of beads are in parentheses and the percentage of total beads in every profile are shown.

Serum reactivity was stratified based on the combination of N, DIL and C1q positive-beads. The results allowed us to discriminate in high or low titer with DIL serum and fixing and non-fixing antibodies after the C1q test. The persistence of high-titer and fixing-complement antibodies, positive results in N, DIL and C1q (N/DIL/C1q) (+/+/+) profile identified specificities at a very high risk in patients and should be considered unacceptable HLA antigens for a potential donor. The absolute number of beads tested is summarized in Fig 2 and a wider range of risk can be defined. Theoretically, all C1q positive beads have a high risk of CDC positive result, whereas other combinations can be stratified in low, moderate and high risk. Interestingly, 310 (36.1%) N positive-beads had negative results after DIL and C1q tests and could be considered as low risk specificities. Inversely, a prozone effect was confirmed in 26 (3.1%) N negative-beads, being positive in both after DIL and C1q tests. The HLA antigens associated with this profile (-/+/+) should be considered as very high risk and, consequently, as prohibited specificities.

### Description of highly-sensitized group

**Fig 3:**
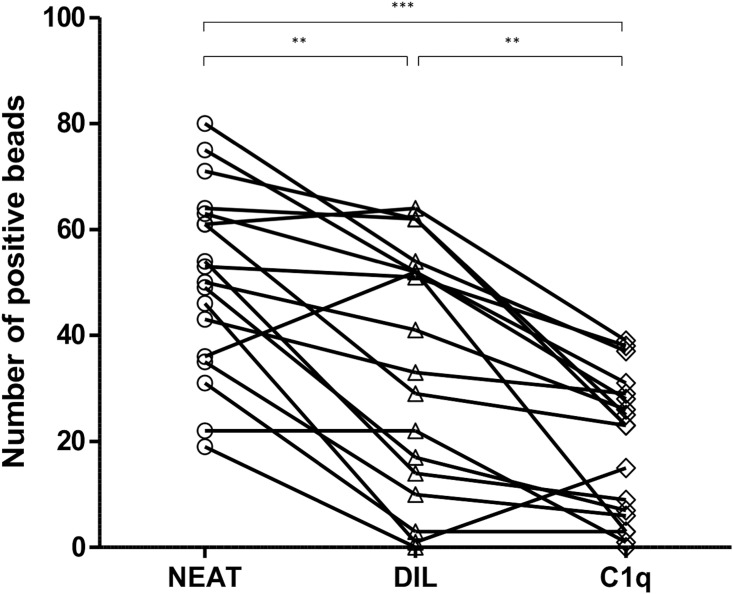
Number of positive beads after study of neat, diluted serum and C1q in highly-sensitized patients. The number of positive beads in 18 highly sensitized patients are depicted after Single Antigen anti-HLA class-I test in neat serum (neat, open circles), in 1/160 diluted serum (DIL, open triangles) and after C1q test (C1q, open diamonds) using the cut off value described in Material and Methods section. Differences of the medians were assessed by Wilcoxon test, (**, p<0.01; ***.p<0.001).

In our cohort of highly sensitized patients, the median of single antigen class-I positive beads was 51.5 beads in neat serum. After 1/160 dilution of the sera, a significant reduction in the median of positive beads was observed, 37.0 beads (Fig 3, p<0.01). In 15 out of 18 highly-sensitized patients (83.3%), a number of unacceptable HLA antigens assigned with N serum with the Single Antigen test were negative after DIL, suggesting that these antibodies could be considered as low-titer antibodies. On the contrary, the prozone effect was observed in 7 out of 18 ptients (38.8%) (at least one bead was considered negative in N serum and positive after DIL). Importantly, the ability to fix the complement was confirmed in 4 patients. The values of raw MFI of beads in N, DIL and C1q test are summarized in S1 Table. Furthermore, C1q analysis revealed a significant decrease in the median of the positive beads (24.0) in highly sensitized patients compared with positive beads after N serum test (Fig 3, p<0.001).

### From the beads to the patients

After the definition of profiles based on bead-results (Fig 2), in our cohort, we identified patients with different profiles based on the results of Single Antigen beads (N/DIL/C1q). In general, the behavior of most beads individually within a patient lowered the level of sensitization. Several examples are shown in S1 Fig. Thus, a highly-sensitized patient, defined only by testing neat serum with Single Antigen assay, could have a low risk profile when most of the beads have negative results after dilution and C1q test (+/-/-) (S1 Fig, panel A). The next risk level could be a patient with moderate risk (+/+/-) when most of the beads are negative in C1q test, despite confirmation of high titer with positive results after dilution (S1 Fig, panel B). The third risk level might be when the beads with anti-HLA antibodies are at low titer but are able to fix the complement (+/-/+), (S1 Fig, panel C). And finally, the most problematic case is a false negative in neat serum after Single Antigen, confirmed in the dilution (prozone effect) and which is able to fix the complement by C1q test (-/+/+) (S1 Fig, panel D and S1 Table).

### Assessment of the risk for different anti-HLA antibodies profiles in complement-dependent cytotoxicity and flow cytometry crossmatches

We have tried to assess the risk of different anti-HLA antibody profiles defined in the previous section. In order to test the low-risk profile (+/-/-), donors with compatible HLA class-I antigens and sera with at least one HLA class-I antigen with (+/-/-) profile were selected. The MFI of N, DIL and C1q of specific donor-HLA antigens are summarized in Table 2 (profile #1). Subsequent CDC and FCXM were performed with negative results (in diluted sera). Furthermore, the moderate risk profile (+/+/-) was designed based on the same fashion (profile #2). Twenty reactions were tested and all CDC were negative, whereas the FCXM rendered a positive result. The high risk (+/-/+) and very high risk profiles (-/+/+) and (+/+/+) were confirmed with positive CDC and FCXM results (profile #3, #4 and #5 respectively, Table 2).

**Table 2:** Assessment of different anti-HLA antibody profiles in highly- sensitized patients.

Profile	Donor type	Profile (Neat/DIL/C1q)	HLA antigen with profile tested	Result Neat (MFI)	Result Dil (MFI)	Result C1q (MFI)	CDC Result	FXCM Result (Ratio^a^ Neat)	FXCM Result (Ratio^a^ Dil)
1	A*02,*03; B*18,*44	+/-/-	A*03:01	Positive (4272)	Negative (134)	Negative (36)	Negative	Positive (9.97)	Negative (1.14)
	A*02,*24; B*27,*44; C*01,*16	+/-/-	A*02:03	Positive (1555)	Negative (47)	Negative (41)	Negative	Positive (3.12)	Negative (0.98)
	A*02,*24; B*27,*44; C*01,*16	+/-/-	B*27:05	Positive (1775)	Negative (491)	Negative (21)	Negative	Positive (4.4)	Negative (1.09)
	A*02,*24; B*27,*44; C*01,*16	+/-/-	B*27:08	Positive (2276)	Negative (601)	Negative (27)	Negative	Positive (4.4)	Negative (1.09)
	A*02,*24; B*27,*44; C*01,*16	+/-/-	B*44:03	Positive (1779)	Negative (413)	Negative (20)	Negative	Positive (4.4)	Negative (1.09)
	A*24,*26; B*07,*51	+/-/-	B*07:02	Positive (3377)	Negative (1166)	Negative (22)	Negative	Positive (1.51)	NP
2	A*03,*29; B*44,*60	+/+/-	A*03:01	Positive (15047)	Positive (18039)	Negative (145)	Negative	Positive (1.71)	Negative (0.89)
	A*03,*29; B*44,*60	+/+/-	A*29:01	Positive (14410)	Positive (14298)	Negative (656)	Negative	Positive (1.71)	Negative (0.89)
	A*03,*29; B*44,*60	+/+/-	A*29:02	Positive (12296)	Positive (14566)	Negative (1067)	Negative	Positive (1.71)	Negative (0.89)
	A*02,*24; B*27,*44; C*01,*16	+/+/-	A*02:01	Positive (14109)	Positive (18281)	Negative (163)	Negative	Positive (5.99)	NP
	A*02,*24; B*27,*44; C*01,*16	+/+/-	A*02:03	Positive (15156)	Positive (18780)	Negative (219)	Negative	Positive (5.99)	NP
	A*02,*24; B*27,*44; C*01,*16	+/+/-	A*02:06	Positive (13062)	Positive (18129)	Negative (339)	Negative	Positive (5.99)	NP
	A*02,*24; B*27,*44; C*01,*16	+/+/-	A*24:02	Positive (14609)	Positive (12901)	Negative (306)	Negative	Positive (5.99)	NP
	A*02,*24; B*27,*44; C*01,*16	+/+/-	A*24:03	Positive (10604)	Positive (13489)	Negative (432)	Negative	Positive (5.99)	NP
	A*02,*24; B*27,*44; C*01,*16	+/+/-	B*27:08	Positive (5353)	Positive (1744)	Negative (455)	Negative	Positive (5.99)	NP
	A*02,*24; B*27,*44; C*01,*16	+/+/-	A*02:01	Positive (3293)	Positive (7030)	Negative (47)	Negative	Positive (3.34)	NP
	A*02,*24; B*27,*44; C*01,*16	+/+/-	A*02:03	Positive (2468)	Positive (5329)	Negative (55)	Negative	Positive (3.34)	NP
	A*02,*24; B*27,*44; C*01,*16	+/+/-	A*02:06	Positive (2615)	Positive (6298)	Negative (39)	Negative	Positive (3.34)	NP
	A*02,*24; B*27,*44; C*01,*16	+/+/-	B*27:05	Positive (4339)	Positive (2566)	Negative (33)	Negative	Positive (3.34)	NP
	A*02,*24; B*27,*44; C*01,*16	+/+/-	B*44:02	Positive (5044)	Positive (9799)	Negative (192)	Negative	Positive (3.34)	NP
	A*24,*26; B*07,*51	+/+/-	A*24:02	Positive (14608)	Positive (12901)	Negative (306)	Negative	Positive (10.16)	NP
	A*24,*26; B*07,*51	+/+/-	A*24:03	Positive (10604)	Positive (13489)	Negative (432)	Negative	Positive (10.16)	NP
	A*24,*26; B*07,*51	+/+/-	A*26:01	Positive (16065)	Positive (17054)	Negative (418)	Negative	Positive (10.16)	NP
	A*24,*26; B*07,*51	+/+/-	B*27:08	Positive (5353)	Positive (1744)	Negative (455)	Negative	Positive (10.16)	NP
	A*24,*26; B*07,*51	+/+/-	B*51:01	Positive (15689)	Positive (16431)	Negative (645)	Negative	Positive (10.16)	NP
	A*24,*26; B*07,*51	+/+/-	B*51:02	Positive (13805)	Positive (16333)	Negative (492)	Negative	Positive (10.16)	NP
3	A*03,*23; B*40,*60	+/-/+	B*40:01	Positive (5119)	Positive (1898)	Positive (20456)	Positive	Positive (2.16)	Negative (1.17)
	A*03,*23; B*40,*60	+/-/+	B*40:02	Positive (4171)	Positive (1686)	Positive (20242)	Positive	Positive (2.16)	Negative (1.17)
	A*24,*26; B*07,*51	+/-/+	A*24:02	Positive (5570)	Positive (1034)	Positive (12202)	Positive	Positive (1.7)	NP
	A*24,*26; B*07,*51	+/-/+	A*24:03	Positive (5910)	Positive (1235)	Positive (11710)	Positive	Positive (1.7)	NP
	A*24,*26; B*07,*51	+/-/+	B*07:02	Positive (4609)	Positive (1434)	Positive (21129)	Positive	Positive (2.08)	NP
4	A*03,*29; B*44,*60	-/+/+	A*03:01	Negative (519)	Positive (9298)	Positive (11801)	Positive	Positive (3.92)	Positive (6.26)
	A*03,*29; B*44,*60	-/+/+	A*29:01	Negative (210)	Positive (9985)	Positive (7132)	Positive	Positive (3.92)	Positive (6.26)
	A*03,*29; B*44,*60	-/+/+	A*29:02	Negative (231)	Positive (9917)	Positive (7341)	Positive	Positive (3.92)	Positive (6.26)
5	A*02,*24; B*27,*44; C*01,*16	+/+/+	B*27:05	Positive (3654)	Positive (6624)	Positive (9842)	Positive	Positive (4.88)	NP
	A*02,*24; B*27,*44; C*01,*16	+/+/+	B*27:08	Positive (3457)	Positive (7032)	Positive (9857)	Positive	Positive (4.88)	NP
	A*02,*24; B*27,*44; C*01,*16	+/+/+	B*27:05	Positive (6007)	Positive (2074)	Positive (18050)	Positive	Positive (4.86)	NP
	A*02,*24; B*27,*44; C*01,*16	+/+/+	B*27:08	Positive (5101)	Positive (1899)	Positive (18113)	Positive	Positive (4.86)	NP
	A*24,*26; B*07,*51	+/+/+	A*24:02	Positive (21360)	Positive (6193)	Positive (16041)	Positive	Positive (6.27)	NP
	A*24,*26; B*07,*51	+/+/+	A*24:03	Positive (22749)	Positive (6798)	Positive (17821)	Positive	Positive (6.27)	NP
	A*24,*26; B*07,*51	+/+/+	A*26:01	Positive (24048)	Positive (11671)	Positive (20357)	Positive	Positive (6.27)	NP
	A*24,*26; B*07,*51	+/+/+	B*51:01	Positive (24914)	Positive (10332)	Positive (12486)	Positive	Positive (6.27)	NP
	A*24,*26; B*07,*51	+/+/+	B*51:02	Positive (25112)	Positive (12142)	Positive (14275)	Positive	Positive (6.27)	NP

^a^ The ratio was calculated: median fluorescence value Serum / median fluorescence value Negative Control. Positive when ratio >1.5

N: neat serum

DIL: Diluted serum

NP: Not performed

### Clinical risk-assessment of the presence of anti-HLA antibodies before kidney transplant recipients with positive FCXM

Five out of the 18 KTR from the study cohort with positive FCXM, but negative CDC, were transplanted. Graft biopsy was performed in four patients to discard ABMR and a protocol biopsy was done/carried out in one recipient. All of them presented class-II donor specific anti-HLA antibodies (DSA) in neat serum. Four of these five patients maintained their high titers of DSA in DIL serum, confirmed by C1q test (Table 3).

**Table 3:** Summary of anti-HLA antibody profile assessment in kidney transplant recipients with negative CDC and positive FCXM.

Case	Donor type	Profile (NEAT/DIL/C1q)	HLA antigen with profile	Neat (MFI)	Dil (MFI)	MFI C1q	CDC	FXCM (Ratio^a^ Neat)	Biopsy indication	Biopsy time after KT	Biopsy result	Plasmapheresis Y/N (number of plasmapheresis)
KTR1	DR*07,13DQ*02,06	+ / - / -	DR13	DR13 Positive (3010)	DR13 Negative (532)	DR13 Negative (390)	Negative	Positive (2.85)	Protocol	13 months	t0, i0, g0, v0, ct0, ci0, cg0, cv0, ptc0, ah0, mm1, C4d0	No
KTR2	DR*13,17DQ*02,06	+ / + / +	DQ6	DQ6 Positive (3655)	DQ6 Positive (3059)	DQ6 Positive (3018)	Negative	Positive (9.55)	Rejection suspiction	2 months	t1, i0, g1, v0, cg0, ci1, ct0, cv1, cg0, ah0, **ptc1**, mm0, **C4d1**	Yes (3)
KTR3	DR*04,16DQ*05,08	+ / + / +	DQ8	DQ8 Positive (11517)	DQ8 Positive (10259)	DQ8 Positive (15002)	Negative	Positive (3.7)	Rejection suspiction	1 month	t0, i0, g2, v0, cg0, ci1, ct1, cv0, **ptc2**, ah0, mm0, **C4d3**	Yes (6)
KTR4	DR*07,08DQ*02,04	- / + / +	DR7 DQ2	DR7 Negative (726) DQ2 Negative (1486)	DR7 Positive (9457) DQ2 Positive (11583)	DR7 Positive (12765) DQ2 Positive (9700)	Negative	Positive (4.91)	Rejection suspiction	3 months	t1, i0, g1, v0, cg1, ci1, ct1, cv0, ah0, mm0, **ptc3**, **C4d1**	Yes (6)
KTR5	DR*07,11DQ*02,03	+ / + / +	DQ2 DQA1*03	DQA1*03 Positive (7709)	DQA1*03 Positive (3206)	DQA1*03 Positive (6054)	Negative	Positive	Rejection suspiction	18 days	t0, i0, g0, v0, ci0, ct0, cg0, cv0, ah2, mm0, **ptc1**, **Cd42**	Yes (7)

KT: Kidney transplantation

KTR: Kidney Transplant Recipient

^a^Ratio FCXM: (median fluorescence channel of neat serum / median fluorescence channel negative control serum); cut off: >2.0

The biopsy confirmed ABMR in the four patients with a high risk profile (1 patient with -/+/+ and 3 patients with +/+/+ profiles) DSA prior transplantation, whereas the patient with low titer and non-fixing complement DSA (+/-/- profile) showed no histological damage in protocol biopsy, without any impairment of graft function. These data confirm the risk-scale for anti-HLA antibodies, proposed and summarized in Table 4.

**Table 4:** Summary of risk-scale proposed based on neat, diluted serum and C1q test results.

Neat	Diluted	C1q	Risk	Number of reactions tested	% of positive CDC	% of positive FCXM
-	-	-	Low	NP	NA	NA
+	-	-	Low	6	0	100*
-	+	-	Moderate	NP	NA	NA
+	+	-	Moderate	20	0	100**
-	-	+	High	NP	NA	NA
+	-	+	High	5	100	100***
-	+	+	Very high	3	100	100
+	+	+	Very high	9	100	100

NP: not performed

NA: not applicable

*5 sera with Negative FCXM after dilution

**3 sera with Negative FCXM after dilution

***2 sera with Negative FCXM after dilution

## Conclusions

The accurate assessment of anti-HLA antibody is mandatory for every tissue-typing laboratory which reports anti-HLA antibodies to identify patients who are eligible for highly-sensitized programs.

There are situations where assigning a positive antigen can be difficult due to low fluorescence signal, and further dilution studies should be performed to rule out a prozone effect[12].

The increased sensitivity of the Luminex test in comparison with earlier ELISA tests has the drawback of using the denatured HLA antigens coated on beads. Besides, the detection of both fixing and non-fixing complement antibodies have led to an increase in false positive results and/or low CDC test, routinely performed in tissue typing laboratories.

Despite the positive correlation between raw MFI data from N and DIL sera shown in this study, a reduction in the number of positive beads was observed after DIL sera in highly-sensitized patients. This demonstrates that in a group of highly-sensitized patients the number of positive beads could be overestimated, possibly due to low titer of anti-HLA antibodies. However, to figure out the potential risk of the low titer of anti-HLA antibodies (negative specificities after DIL) we suggest performing C1q assay. The complement-binding assay is useful in monitoring kidney transplant recipients at risk for allograft loss[13]. The readout of the possible combinations proposed here opens different scenarios other than just positive or negative specificities. We propose a gradual risk of the specificities derived from the results in N/DIL/C1q sera, summarized in Fig 2 and Table 4.

In order to assess the low risk of the specificities of sera with positive N results, but negative after DIL and C1q, we performed CDC and FCXM tests with PBMC with reactive HLA specificities. These sera resulted negative in the CDC, confirming the low risk specificities of the profile (+/-/-) (Table 2).

In contrast, in our cohort we observed a high rate of highly-sensitized patients (22.2%) with at least one HLA class-I specificity with prozone effect and complement-fixing antibodies (S1 Table). The detection of this phenomenon is crucial for accurate definition of an anti-HLA antibody profile. This is especially important to avoid negative vXM results that would render a final positive CDC crossmatch. The limitation of this observation is based on a single serum test, but patients on the renal waiting list are frequently monitored and this situation could be detected in other sera. Recently, the complement interference as a cause of fluctuations of serum anti-HLA antibody strength has been demonstrated[12,14].

As proof of concept, five patients from our cohort could be transplanted despite their hypersensitized status. In four of them, ABMR was demonstrated by biopsy and all of them showed a high risk profile as defined here. On the contrary, the one patient with a low profile who was transplanted from our cohort did not suffer ABMR or graft loss. Due to the scarce number of highly-sensitized patients finally transplanted in our institution, where only 5 patients were tested, a larger number of highly-sensitized patients should be monitored to confirm these findings.

A limitation of the present study was the use of only class-I coated beads for simplicity. Similar results were observed in class-II antigens coated beads (S1 File). However the interpretation of the anti-HLA antibody profile is more complex, based on reactions against the alpha and/or beta chains (DQA, DQB, DPA and/or DPB antigens) and consequently the assessment of the risk profile results are more difficult to demonstrate. Moreover, in our cohort of kidney transplant recipients, all anti-HLA antibody profiles tested were against class-II antigens with similar findings (Table 3).

We consider maintaining HLA-antigens with the (+/+/-) antibody profile (moderate risk) as unacceptable. Non-fixing IgG4 or IgG2 anti-HLA antibodies might sustain this profile, although in the present study we did not perform specific IgG subclass to confirm it. Irrespectively of the IgG subclass, the IgG class switch cannot be ruled out and the change to a complement fixing IgG1 or IgG3 would increase the risk of a rejection event. Indeed, the recommendation from recent guidelines is to assign HLA mismatches from previous transplants as unacceptable antigens, despite the lack of complement binding antibodies[8].

The highly-sensitized patients spend prolonged periods of time on the waiting list, and the accurate definition of acceptable HLA antigens with low risk should be a priority in order to minimize the time waiting for an organ to be able to be allocated.

The better definition of anti-HLA antibody profile after dilution and C1q test could open up new opportunities in those highly-sensitized patients with a high number of HLA specificities at low titers and non-complement fixing antibodies but would also allow identification of the prozone effect with undesirable consequences in organ allocation systems based on vXM.

## Supporting information

S1 Table Highly sensitized patients with prozone beads detected. (DOC)

S1 Fig Representative patterns of anti-HLA antibody regarding positive beads after neat, dilution and C1q test. Tridimentional scatterplots of MFI values after neat serum (z-axis), 1/160 dilution (y-axis) and C1q (x-axis) test. The colour scale based on MFI from C1q test represents low level (blue), intermediate levels (red-orange) and high MFI levels (yellow). The risk profile showed after neat serum, 1/160 dilution and C1q tes (neat/Dil/C1q) are A) low risk profile (+/-/-), B) moderate risk (+/+/-), C) high risk (+/-/+) and D) very high risk (-/+/+). The Excel macro used (http://www.doka.ch/Excel3Dscatterplot.htm).
(TIFF)

S1 File Raw data of Single Antigen beads for class-I and class-II antigens after assessment of net, diluted serum and C1q test. (XLSX)

## Abbreviations

ABMR: antibody-mediated rejection
C1q: C1q test
CDC: complement-dependent cytotoxicity
cPRA: calculated-panel reactive of antibodies
DIL: diluted serum
DSA: donor-specific anti-HLA antibodies
FCXM: flow cytometry crossmatches
MFI: mean fluorescence intensity
N: neat serum
PBMCs: peripheral blood mononuclear cells
PBS: phosphate buffer saline
SPA: Solid phase assay
vXM: virtual crossmatching

## References

[1] Minucci PB, Grimaldi V, Casamassimi A, Cacciatore F, Sommese L, Picascia A, et al Methodologies for anti-HLA antibody screening in patients awaiting kidney transplant: a comparative study. Experimental and clinical transplantation: official journal of the Middle East Society for Organ Transplantation. 2011;9(6):381–6. 22142045

[2] Morris GP, Phelan DL, Jendrisak MD, Mohanakumar T. Virtual crossmatch by identification of donor-specific anti-human leukocyte antigen antibodies by solid-phase immunoassay: a 30-month analysis in living donor kidney transplantation. Human immunology. 2010;71(3):268–73. 20074605 10.1016/j.humimm.2010.01.003

[3] Jackson AM. The Virtual Crossmatch: An Essential Tool for Transplanting Sensitized Patients. Clinical transplants. 2014:131–6. 26281137

[4] Tait BD, Hudson F, Brewin G, Cantwell L, Holdsworth R. Solid phase HLA antibody detection technology--challenges in interpretation. Tissue antigens. 2010;76(2):87–95. 10.1111/j.1399-0039.2010.01486.x 20403141

[5] Bray RA, Gebel HM. Strategies for human leukocyte antigen antibody detection. Current opinion in organ transplantation. 2009;14(4):392–7. 19610172 10.1097/mot.0b013e32832d31c7

[6] Endres RO, Kleinman SH, Carrick DM, Steele WR, Wright DJ, Norris PJ, et al Identification of specificities of antibodies against human leukocyte antigens in blood donors. Transfusion. 2010;50(8):1749–60. 10.1111/j.1537-2995.2010.02589.x 20158682 PMC3061817

[7] Visentin J, Vigata M, Daburon S, Contin-Bordes C, Fremeaux-Bacchi V, Dromer C, et al Deciphering complement interference in anti-human leukocyte antigen antibody detection with flow beads assays. Transplantation. 2014;98(6):625–31. 10.1097/TP.0000000000000315 25058376

[8] Tait BD, Susal C, Gebel HM, Nickerson PW, Zachary AA, Claas FH, et al Consensus guidelines on the testing and clinical management issues associated with HLA and non-HLA antibodies in transplantation. Transplantation. 2013;95(1):19–47. 10.1097/TP.0b013e31827a19cc 23238534

[9] Tambur AR, Herrera ND, Haarberg KM, Cusick MF, Gordon RA, Leventhal JR, et al Assessing Antibody Strength: Comparison of MFI, C1q, and Titer Information. American journal of transplantation: official journal of the American Society of Transplantation and the American Society of Transplant Surgeons. 2015;15(9):2421–30. 10.1111/ajt.13295 25930984

[10] Visentin J, Guidicelli G, Taupin JL. Assessing HLA Antibody Strength: Have We Thought About Everything? American journal of transplantation: official journal of the American Society of Transplantation and the American Society of Transplant Surgeons. 2015;15(12):3271. 10.1111/ajt.13452 26361370

[11] Tambur AR, Ramon DS, Kaufman DB, Friedewald J, Luo X, Ho B, et al Perception versus reality?: Virtual crossmatch--how to overcome some of the technical and logistic limitations. American journal of transplantation: official journal of the American Society of Transplantation and the American Society of Transplant Surgeons. 2009;9(8):1886–93. 10.1111/j.1600-6143.2009.02724.x PMC4094140 19563341

[12] Berth M. Complement interference is not the same as a prozone phenomenon. American journal of transplantation: official journal of the American Society of Transplantation and the American Society of Transplant Surgeons. 2015. 10.1111/ajt.13650 26639837

[13] Loupy A, Lefaucheur C, Vernerey D, Prugger C, Duong van Huyen JP, Mooney N, et al Complement-binding anti-HLA antibodies and kidney allograft survival. N Engl J Med. 2013;369:1215–1226. 10.1056/NEJMoa1302506 24066742

[14] Guidicelli G, Anies G, Bachelet T, Dubois V, Moreau JF, Merville P, et al The complement interference phenomenon as a cause for sharp fluctuations of serum anti-HLA antibody strength in kidney transplant patients. Transplant immunology. 2013;29(1–4):17–21. 10.1016/j.trim.2013.09.005 24056164

