# Supplementary material for: Improvement in the definition of anti-HLA antibody profile in highly sensitized patients
Source: PLoS One. 2017 Feb 3;12(2):e0171463. doi: 10.1371/journal.pone.0171463 (PMC5291387; doi:10.1371/journal.pone.0171463)
Supplement: S1 Table — (DOC) [file pone.0171463.s001.doc]

**Suppl Table 1**. Highly sensitized patients with prozone beads detected.

| Case number | Serologic | Allelic | NEAT Raw MFI | DIL Raw MFI | C1q Raw MFI |
| --- | --- | --- | --- | --- | --- |
| 1 | B7 | B*07:02 | 2017,4 | 19535,98 | 15094,01 |
|  | B81 | B*81:01 | 2822,86 | 18896,88 | 12138,4 |
| 2 | A30 | A*30:01 | 1104,88 | 16937,04 | 14832,8 |
|  | A31 | A*31:01 | 625,78 | 18079,19 | 14392,57 |
| 3 | A1 | A*01:01 | 1102,58 | 16383,91 | 10015,93 |
| 4 | A3 | A*03:01 | 518,54 | 11800,62 | 9298,35 |
|  | A11 | A*11:02 | 567,23 | 12553,58 | 7986,71 |
|  | A25 | A*25:01 | 639,55 | 11434,32 | 6665,54 |
|  | A26 | A*26:01 | 714,47 | 13027,29 | 8743,57 |
|  | A29 | A*29:01 | 209,65 | 7131,56 | 9985,43 |
|  | A29 | A*29:02 | 231,12 | 7340,57 | 9916,65 |
|  | A30 | A*30:01 | 386,25 | 8108,42 | 9094,2 |
|  | A30 | A*30:02 | 632,97 | 10091,13 | 10102,32 |
|  | A31 | A*31:01 | 222,59 | 6457,67 | 9266,38 |
|  | A32 | A*32:01 | 363,84 | 10203,32 | 8660,74 |
|  | A33 | A*33:01 | 491,77 | 7901,03 | 9413,3 |
|  | A34 | A*34:02 | 697,47 | 8726,74 | 9389,97 |
|  | A43 | A*43:01 | 616,55 | 11984,03 | 9440,49 |
|  | A66 | A*66:02 | 681,8 | 12211,95 | 8179,43 |
|  | A74 | A*74:01 | 410,11 | 11327,07 | 8607,9 |
|  | A80 | A*80:01 | 604,39 | 12998,89 | 8762,49 |
|  | B67 | B*67:01 | 646,78 | 9356,45 | 8733,72 |
|  | A33 | A*33:03 | 360,4 | 8255,95 | 9054,68 |

MFI: mean fluorescence intensity
